# (*E*)-1-(4-Decyl­oxyphen­yl)-3-(2-hy­droxy­phen­yl)prop-2-en-1-one

**DOI:** 10.1107/S160053681203872X

**Published:** 2012-09-15

**Authors:** Zainab Ngaini, Siti Muhaini Haris Fadzillah, Hasnain Hussain, Ibrahim Abdul Razak, Safra Izuani Jama Asik

**Affiliations:** a Department of Chemistry, Faculty of Resource Science and Technology, Universiti Malaysia Sarawak, 94300 Kota Samarahan, Sarawak, Malaysia; b Department of Molecular Biology, Faculty of Resource Science and Technology, Universiti Malaysia Sarawak, 94300 Kota Samarahan, Sarawak, Malaysia; c School of Physics, Universiti Sains Malaysia, 11800 USM, Penang, Malaysia

## Abstract

In the title compound, C_25_H_32_O_3_, the enone group adopts an *s-cis* conformation. The alk­oxy chain is in an all-*trans* conformation. The dihedral angle between the benzene rings is 7.86 (5)°. In the crystal, mol­ecules are connected by pairs of O—H⋯O hydrogen bonds, forming inversion dimers and giving *R*_2_^2^(10) rings. Within these dimers, weak C—H⋯O hydrogen bonds form two *R*_2_^2^(7) rings. In the crystal, the approximately planar mol­ecules [largest deviation for an atom being 0.4737 (12) Å for the terminal C atom of the alk­oxy chain] are arranged in sheets parallel to (20-1). Weak C—H⋯π inter­actions are also observed.

## Related literature

For the biological properties of chalcone derivatives, see: Bhat *et al.* (2005 ▶); Xue *et al.* (2004 ▶); Won *et al.* (2005 ▶); Zhao *et al.* (2005 ▶); Satyanarayana *et al.* (2004 ▶). For related structures, see: Razak *et al.* (2009 ▶); Ngaini *et al.* (2010 ▶, 2011 ▶). For graph-set theory, see: Bernstein *et al.* (1995 ▶). For the stability of the temperature controller used in the data collection, see: Cosier & Glazer (1986) ▶. For standard bond-length data, see: Allen *et al.* (1987 ▶).
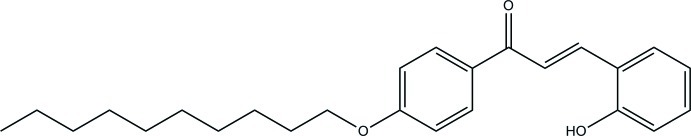


## Experimental

### 

#### Crystal data

C_25_H_32_O_3_
*M* *_r_* = 380.51
Triclinic, 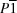

*a* = 8.6674 (13) Å
*b* = 10.9865 (17) Å
*c* = 12.1352 (19) Å
α = 74.405 (3)°
β = 72.891 (3)°
γ = 85.981 (4)°
*V* = 1063.7 (3) Å^3^
*Z* = 2
Mo *K*α radiation
μ = 0.08 mm^−1^
*T* = 100 K
0.62 × 0.15 × 0.14 mm

#### Data collection

Bruker APEXII DUO CCD area-detector diffractometer
Absorption correction: multi-scan (*SADABS*; Bruker, 2009 ▶) *T*_min_ = 0.955, *T*_max_ = 0.990
30192 measured reflections
8434 independent reflections
6213 reflections with *I* > 2σ(*I*)
*R*_int_ = 0.034

#### Refinement

*R*[*F* ^2^ > 2σ(*F* ^2^)] = 0.050
*wR*(*F* ^2^) = 0.160
*S* = 1.02
8434 reflections
258 parameters
H atoms treated by a mixture of independent and constrained refinement
Δρ_max_ = 0.63 e Å^−3^
Δρ_min_ = −0.20 e Å^−3^

### 

Data collection: *APEX2* (Bruker, 2009 ▶); cell refinement: *SAINT* (Bruker, 2009 ▶); data reduction: *SAINT*; program(s) used to solve structure: *SHELXTL* (Sheldrick, 2008 ▶); program(s) used to refine structure: *SHELXTL*; molecular graphics: *SHELXTL*; software used to prepare material for publication: *SHELXTL* and *PLATON* (Spek, 2009 ▶).

## Supplementary Material

Crystal structure: contains datablock(s) global, I. DOI: 10.1107/S160053681203872X/lh5525sup1.cif

Structure factors: contains datablock(s) I. DOI: 10.1107/S160053681203872X/lh5525Isup2.hkl

Supplementary material file. DOI: 10.1107/S160053681203872X/lh5525Isup3.cml

Additional supplementary materials:  crystallographic information; 3D view; checkCIF report

## Figures and Tables

**Table 1:** Hydrogen-bond geometry (Å, °) *Cg*1 is the centroid of the C10–C15 ring.

*D*—H⋯*A*	*D*—H	H⋯*A*	*D*⋯*A*	*D*—H⋯*A*
O1—H1*O*1⋯O2^i^	0.939 (18)	1.789 (18)	2.6867 (12)	159.0 (16)
C7—H7*A*⋯O1^i^	0.93	2.31	3.2169 (13)	164
C22—H22*A*⋯*Cg*1^ii^	0.97	2.85	3.6887 (12)	146

Symmetry codes: (i) 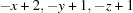; (ii) 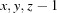.

**Fig. 1.**
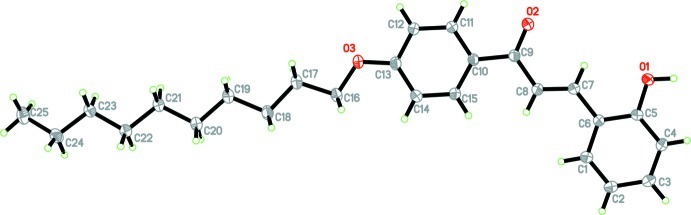
The molecular structure of the title compound, showing 50% probability displacement ellipsoids.

**Fig. 2.**
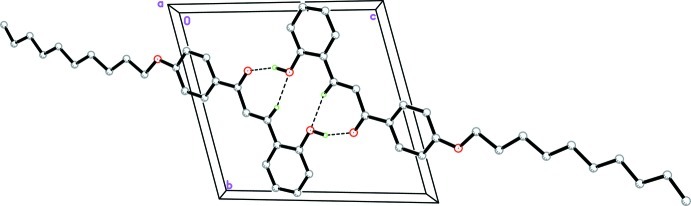
A pair inversion related molecules connected by hydrogen bonds (dashed lines).

**Fig. 3.**
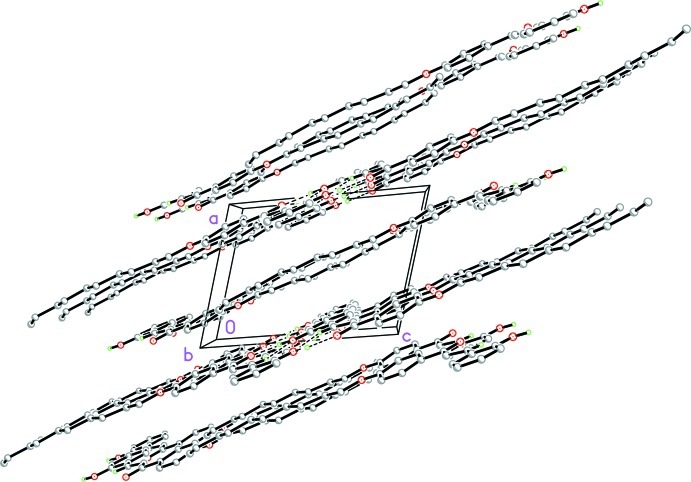
The crystal packing, viewed along the b-axis, showing the molecules in pairs, arranged into sheets parallel to (201) plane. Hydrogen bonds are shown as dashed lines. Only H atoms involved in hydrogen bonds are shown.

## supplementary crystallographic information

### Comment

Chalcones have displayed an impressive array of biological activities and are extensively reported (Xue *et al.*, 2004; Bhat *et al.*, 2005; Won *et al.*, 2005; Zhao *et al.*, 2005 and Satyanarayana *et al.*, 2004). Here in, we report the crystal structure of the title compound (I).

In (I), Fig. 1, the bond lengths observed are comparable with standard reported values (Allen *et al.*, 1987). The enone (O2/C7–C9) moiety adopts a *s-cis* conformation with a torsion angle of -6.41 (15)°. The mean plane through the enone (O2/C7–C9) moiety form dihedral angles of 2.80 (6) and 7.26 (6)° respectively with the benzene (C1–C6 and C10–C15) rings. The dihedral angle between the two benzene rings is 7.86 (5)°.

An observed widening of the C1–C6–C7 and C6–C7–C8 angles of 122.85 (8) and 126.23 (8)° respectively, may be the consequence of the short contact between H1A and H8A (2.19 Å). Similarly, the slight distortion of the O3–C13–C14 angle to 125.18 (9)° may be the result of a close H14A and H16B (2.37 Å) contact. The geometric parameters are consistent to those observed in closely related structures (Razak *et al.*, 2009; Ngaini *et al.*, 2010; Ngaini *et al.*, 2011).

The alkoxy chain adopts an all *trans* conformation with the difference from the ideal values of the torsion angles ranging from 0.98 (7)° to 7.98 (9)°. The C16–O3–C13–C14 torsion angle of 4.73 (13)° indicate that atoms C16 and O3 and the attached benzene ring are approximately co-planar. The alkoxy chain appears to deviate from co-planarity with the ring for atoms further away i.e. C24 and C25. The dihedral angle between the least-square plane through atoms O3/C16–C25 [maximum deviation = 0.2507 (10)Å at C25] and the attached benzene ring is 15.20 (5)°.

In the crystal (Fig. 2), molecules are connected by a pair of O1—H1O1···O2^i^ and a pair of weak C7—H7A···O1^i^ hydrogen bonds to form a *R*^2^_2_(10) ring and two *R*^2^_2_(7) rings within inversion dimers. These dimers are arranged into sheets parallel to (201). Weak C—H···π interactions (Table 1) are also observed.

### Experimental

A mixture of 2-hydroxybenzaldehyde (2.44 ml, 20 mmol), 4-decyloxyacetophenone (6.65 g, 20 mmol) and KOH (4.04 g, 72 mmol) in methanol (60 ml) was heated at reflux for 12 h. The reaction was cooled to room temperature and acidified with cold diluted HCl (2N). After redissolving in hexane followed by few days of slow evaporation, crystals were collected.

### Refinement

The O-bound H atom was located in a difference Fourier map and refined freely with O–H = 0.943 (17) Å. The remaining H atoms were placed in calculated positions with C–H = 0.93–0.97 Å. The *U*_iso_ values were constrained to be 1.5*U*_eq_ (methyl-H atom) and 1.2*U*_eq_ (other H atoms). The rotating model group was applied for the methyl group.

### Crystal data

**Table:**

C_25_H_32_O_3_	*Z* = 2
*M**_r_* = 380.51	*F*(000) = 412
Triclinic, *P*1	*D*_x_ = 1.188 Mg m^−^^3^
Hall symbol: -P 1	Mo *K*α radiation, λ = 0.71073 Å
*a* = 8.6674 (13) Å	Cell parameters from 7111 reflections
*b* = 10.9865 (17) Å	θ = 2.5–33.7°
*c* = 12.1352 (19) Å	µ = 0.08 mm^−^^1^
α = 74.405 (3)°	*T* = 100 K
β = 72.891 (3)°	Block, yellow
γ = 85.981 (4)°	0.62 × 0.15 × 0.14 mm
*V* = 1063.7 (3) Å^3^	

### Data collection

**Table:**

Bruker APEXII DUO CCD area-detector diffractometer	8434 independent reflections
Radiation source: fine-focus sealed tube	6213 reflections with *I* > 2σ(*I*)
Graphite monochromator	*R*_int_ = 0.034
φ and ω scans	θ_max_ = 33.9°, θ_min_ = 2.5°
Absorption correction: multi-scan (*SADABS*; Bruker, 2009)	*h* = −13→13
*T*_min_ = 0.955, *T*_max_ = 0.990	*k* = −17→17
30192 measured reflections	*l* = −18→18

### Refinement

**Table:**

Refinement on *F*^2^	Primary atom site location: structure-invariant direct methods
Least-squares matrix: full	Secondary atom site location: difference Fourier map
*R*[*F*^2^ > 2σ(*F*^2^)] = 0.050	Hydrogen site location: inferred from neighbouring sites
*wR*(*F*^2^) = 0.160	H atoms treated by a mixture of independent and constrained refinement
*S* = 1.02	*w* = 1/[σ^2^(*F*_o_^2^) + (0.0957*P*)^2^ + 0.086*P*] where *P* = (*F*_o_^2^ + 2*F*_c_^2^)/3
8434 reflections	(Δ/σ)_max_ < 0.001
258 parameters	Δρ_max_ = 0.63 e Å^−^^3^
0 restraints	Δρ_min_ = −0.20 e Å^−^^3^

### Special details

**Table:**

Experimental. The crystal was placed in the cold stream of an Oxford Cryosystems Cobra open-flow nitrogen cryostat (Cosier & Glazer, 1986) operating at 100.0 (1) K.
Geometry. All esds (except the esd in the dihedral angle between two l.s. planes) are estimated using the full covariance matrix. The cell esds are taken into account individually in the estimation of esds in distances, angles and torsion angles; correlations between esds in cell parameters are only used when they are defined by crystal symmetry. An approximate (isotropic) treatment of cell esds is used for estimating esds involving l.s. planes.
Refinement. Refinement of F^2^ against ALL reflections. The weighted R-factor wR and goodness of fit S are based on F^2^, conventional R-factors R are based on F, with F set to zero for negative F^2^. The threshold expression of F^2^ > 2sigma(F^2^) is used only for calculating R-factors(gt) etc. and is not relevant to the choice of reflections for refinement. R-factors based on F^2^ are statistically about twice as large as those based on F, and R- factors based on ALL data will be even larger.

### Fractional atomic coordinates and isotropic or equivalent isotropic displacement parameters (Å^2^)

**Table:**

	*x*	*y*	*z*	*U*_iso_*/*U*_eq_	
O1	0.97173 (10)	0.64655 (7)	0.51189 (7)	0.03236 (17)	
O2	0.94280 (8)	0.34433 (6)	0.29484 (6)	0.02528 (14)	
O3	0.72670 (9)	0.27611 (6)	−0.13495 (6)	0.02605 (15)	
C1	0.83637 (12)	0.82610 (8)	0.26041 (8)	0.02406 (17)	
H1A	0.8087	0.8127	0.1961	0.029*	
C2	0.82611 (13)	0.94641 (9)	0.27668 (9)	0.0290 (2)	
H2A	0.7910	1.0128	0.2241	0.035*	
C3	0.86846 (12)	0.96808 (9)	0.37210 (9)	0.02815 (19)	
H3A	0.8630	1.0493	0.3825	0.034*	
C4	0.91859 (11)	0.86906 (8)	0.45150 (9)	0.02482 (17)	
H4A	0.9466	0.8836	0.5152	0.030*	
C5	0.92699 (11)	0.74707 (8)	0.43560 (8)	0.02130 (16)	
C6	0.88752 (10)	0.72389 (8)	0.33845 (7)	0.01911 (15)	
C7	0.89920 (10)	0.59575 (8)	0.32398 (8)	0.02062 (16)	
H7A	0.9260	0.5326	0.3836	0.025*	
C8	0.87499 (11)	0.56017 (8)	0.23288 (8)	0.02137 (16)	
H8A	0.8464	0.6213	0.1727	0.026*	
C9	0.89166 (10)	0.42876 (8)	0.22394 (7)	0.01902 (15)	
C10	0.84626 (10)	0.39669 (8)	0.12615 (7)	0.01838 (15)	
C11	0.87712 (10)	0.27355 (8)	0.11183 (8)	0.02067 (16)	
H11A	0.9258	0.2158	0.1626	0.025*	
C12	0.83636 (11)	0.23708 (8)	0.02362 (8)	0.02239 (16)	
H12A	0.8581	0.1555	0.0151	0.027*	
C13	0.76235 (10)	0.32268 (8)	−0.05314 (7)	0.02090 (16)	
C14	0.73151 (11)	0.44598 (8)	−0.04146 (8)	0.02183 (16)	
H14A	0.6834	0.5036	−0.0927	0.026*	
C15	0.77373 (10)	0.48127 (8)	0.04772 (7)	0.02078 (16)	
H15A	0.7532	0.5633	0.0554	0.025*	
C16	0.66227 (11)	0.35667 (9)	−0.22515 (8)	0.02424 (17)	
H16A	0.7379	0.4242	−0.2752	0.029*	
H16B	0.5613	0.3935	−0.1892	0.029*	
C17	0.63573 (12)	0.27105 (9)	−0.29722 (8)	0.02506 (17)	
H17A	0.5556	0.2074	−0.2455	0.030*	
H17B	0.7358	0.2277	−0.3235	0.030*	
C18	0.58040 (11)	0.33829 (9)	−0.40639 (8)	0.02417 (17)	
H18A	0.4746	0.3743	−0.3809	0.029*	
H18B	0.6551	0.4066	−0.4560	0.029*	
C19	0.57271 (12)	0.24580 (9)	−0.47838 (8)	0.02537 (18)	
H19A	0.5075	0.1735	−0.4251	0.030*	
H19B	0.6809	0.2157	−0.5082	0.030*	
C20	0.50411 (11)	0.29821 (9)	−0.58370 (8)	0.02291 (16)	
H20A	0.3978	0.3323	−0.5557	0.027*	
H20B	0.5730	0.3666	−0.6408	0.027*	
C21	0.49168 (11)	0.19692 (9)	−0.64525 (8)	0.02409 (17)	
H21A	0.4292	0.1263	−0.5861	0.029*	
H21B	0.5993	0.1665	−0.6763	0.029*	
C22	0.41473 (11)	0.24020 (8)	−0.74698 (8)	0.02339 (17)	
H22A	0.4848	0.3026	−0.8115	0.028*	
H22B	0.3129	0.2806	−0.7190	0.028*	
C23	0.38450 (12)	0.13207 (9)	−0.79502 (8)	0.02553 (18)	
H23A	0.4858	0.0898	−0.8200	0.031*	
H23B	0.3116	0.0712	−0.7310	0.031*	
C24	0.31312 (12)	0.17379 (10)	−0.89953 (9)	0.02908 (19)	
H24A	0.3923	0.2250	−0.9676	0.035*	
H24B	0.2199	0.2261	−0.8785	0.035*	
C25	0.26215 (14)	0.06325 (11)	−0.93513 (10)	0.0364 (2)	
H25A	0.2134	0.0949	−0.9987	0.055*	
H25B	0.1857	0.0109	−0.8676	0.055*	
H25C	0.3552	0.0143	−0.9615	0.055*	
H1O1	0.9937 (19)	0.6709 (15)	0.5747 (15)	0.054 (4)*	

### Atomic displacement parameters (Å^2^)

**Table:**

	*U*^11^	*U*^22^	*U*^33^	*U*^12^	*U*^13^	*U*^23^
O1	0.0556 (5)	0.0212 (3)	0.0321 (4)	0.0096 (3)	−0.0303 (3)	−0.0091 (3)
O2	0.0347 (3)	0.0214 (3)	0.0252 (3)	0.0039 (2)	−0.0166 (3)	−0.0073 (2)
O3	0.0373 (4)	0.0246 (3)	0.0235 (3)	0.0037 (3)	−0.0170 (3)	−0.0099 (2)
C1	0.0322 (4)	0.0187 (4)	0.0236 (4)	0.0014 (3)	−0.0130 (3)	−0.0042 (3)
C2	0.0410 (5)	0.0178 (4)	0.0316 (5)	0.0032 (3)	−0.0178 (4)	−0.0049 (3)
C3	0.0368 (5)	0.0178 (4)	0.0343 (5)	0.0028 (3)	−0.0164 (4)	−0.0082 (3)
C4	0.0310 (4)	0.0204 (4)	0.0287 (4)	0.0026 (3)	−0.0143 (3)	−0.0100 (3)
C5	0.0247 (4)	0.0178 (3)	0.0240 (4)	0.0031 (3)	−0.0112 (3)	−0.0058 (3)
C6	0.0220 (4)	0.0171 (3)	0.0198 (3)	0.0004 (3)	−0.0084 (3)	−0.0047 (3)
C7	0.0249 (4)	0.0171 (3)	0.0216 (4)	0.0016 (3)	−0.0092 (3)	−0.0055 (3)
C8	0.0273 (4)	0.0179 (3)	0.0209 (4)	−0.0003 (3)	−0.0104 (3)	−0.0045 (3)
C9	0.0203 (3)	0.0191 (3)	0.0188 (3)	−0.0006 (3)	−0.0068 (3)	−0.0052 (3)
C10	0.0205 (3)	0.0181 (3)	0.0172 (3)	−0.0011 (3)	−0.0063 (3)	−0.0044 (3)
C11	0.0247 (4)	0.0188 (3)	0.0207 (4)	0.0014 (3)	−0.0100 (3)	−0.0054 (3)
C12	0.0291 (4)	0.0196 (4)	0.0220 (4)	0.0021 (3)	−0.0114 (3)	−0.0073 (3)
C13	0.0240 (4)	0.0225 (4)	0.0182 (3)	−0.0006 (3)	−0.0076 (3)	−0.0066 (3)
C14	0.0259 (4)	0.0211 (4)	0.0206 (4)	0.0019 (3)	−0.0102 (3)	−0.0055 (3)
C15	0.0252 (4)	0.0187 (3)	0.0197 (3)	0.0007 (3)	−0.0083 (3)	−0.0052 (3)
C16	0.0285 (4)	0.0271 (4)	0.0210 (4)	0.0023 (3)	−0.0114 (3)	−0.0085 (3)
C17	0.0295 (4)	0.0281 (4)	0.0221 (4)	−0.0002 (3)	−0.0114 (3)	−0.0093 (3)
C18	0.0263 (4)	0.0281 (4)	0.0218 (4)	0.0012 (3)	−0.0100 (3)	−0.0093 (3)
C19	0.0302 (4)	0.0279 (4)	0.0216 (4)	0.0011 (3)	−0.0103 (3)	−0.0092 (3)
C20	0.0246 (4)	0.0263 (4)	0.0197 (4)	−0.0002 (3)	−0.0079 (3)	−0.0073 (3)
C21	0.0289 (4)	0.0260 (4)	0.0207 (4)	0.0016 (3)	−0.0110 (3)	−0.0077 (3)
C22	0.0264 (4)	0.0243 (4)	0.0219 (4)	0.0015 (3)	−0.0102 (3)	−0.0068 (3)
C23	0.0316 (4)	0.0254 (4)	0.0239 (4)	0.0023 (3)	−0.0144 (3)	−0.0070 (3)
C24	0.0330 (5)	0.0309 (5)	0.0279 (4)	0.0006 (4)	−0.0176 (4)	−0.0056 (4)
C25	0.0427 (6)	0.0421 (6)	0.0349 (5)	0.0011 (5)	−0.0217 (4)	−0.0156 (5)

### Geometric parameters (Å, º)

**Table:**

O1—C5	1.3523 (10)	C16—C17	1.5146 (12)
O1—H1O1	0.943 (17)	C16—H16A	0.9700
O2—C9	1.2389 (10)	C16—H16B	0.9700
O3—C13	1.3485 (10)	C17—C18	1.5260 (13)
O3—C16	1.4363 (11)	C17—H17A	0.9700
C1—C2	1.3811 (13)	C17—H17B	0.9700
C1—C6	1.4000 (12)	C18—C19	1.5236 (12)
C1—H1A	0.9300	C18—H18A	0.9700
C2—C3	1.3938 (14)	C18—H18B	0.9700
C2—H2A	0.9300	C19—C20	1.5239 (13)
C3—C4	1.3846 (13)	C19—H19A	0.9700
C3—H3A	0.9300	C19—H19B	0.9700
C4—C5	1.3985 (12)	C20—C21	1.5228 (12)
C4—H4A	0.9300	C20—H20A	0.9700
C5—C6	1.4080 (12)	C20—H20B	0.9700
C6—C7	1.4579 (11)	C21—C22	1.5229 (12)
C7—C8	1.3414 (12)	C21—H21A	0.9700
C7—H7A	0.9300	C21—H21B	0.9700
C8—C9	1.4706 (12)	C22—C23	1.5250 (13)
C8—H8A	0.9300	C22—H22A	0.9700
C9—C10	1.4854 (11)	C22—H22B	0.9700
C10—C15	1.3998 (11)	C23—C24	1.5201 (13)
C10—C11	1.4072 (12)	C23—H23A	0.9700
C11—C12	1.3800 (12)	C23—H23B	0.9700
C11—H11A	0.9300	C24—C25	1.5252 (15)
C12—C13	1.3999 (12)	C24—H24A	0.9700
C12—H12A	0.9300	C24—H24B	0.9700
C13—C14	1.3988 (12)	C25—H25A	0.9600
C14—C15	1.3906 (12)	C25—H25B	0.9600
C14—H14A	0.9300	C25—H25C	0.9600
C15—H15A	0.9300		
			
C5—O1—H1O1	110.9 (10)	C16—C17—H17A	108.6
C13—O3—C16	120.53 (7)	C18—C17—H17A	108.6
C2—C1—C6	121.51 (9)	C16—C17—H17B	108.6
C2—C1—H1A	119.2	C18—C17—H17B	108.6
C6—C1—H1A	119.2	H17A—C17—H17B	107.6
C1—C2—C3	119.86 (9)	C19—C18—C17	110.31 (8)
C1—C2—H2A	120.1	C19—C18—H18A	109.6
C3—C2—H2A	120.1	C17—C18—H18A	109.6
C4—C3—C2	120.20 (9)	C19—C18—H18B	109.6
C4—C3—H3A	119.9	C17—C18—H18B	109.6
C2—C3—H3A	119.9	H18A—C18—H18B	108.1
C3—C4—C5	119.80 (8)	C18—C19—C20	115.59 (8)
C3—C4—H4A	120.1	C18—C19—H19A	108.4
C5—C4—H4A	120.1	C20—C19—H19A	108.4
O1—C5—C4	122.25 (8)	C18—C19—H19B	108.4
O1—C5—C6	116.99 (8)	C20—C19—H19B	108.4
C4—C5—C6	120.76 (8)	H19A—C19—H19B	107.4
C1—C6—C5	117.86 (8)	C21—C20—C19	111.70 (8)
C1—C6—C7	122.86 (8)	C21—C20—H20A	109.3
C5—C6—C7	119.27 (7)	C19—C20—H20A	109.3
C8—C7—C6	126.24 (8)	C21—C20—H20B	109.3
C8—C7—H7A	116.9	C19—C20—H20B	109.3
C6—C7—H7A	116.9	H20A—C20—H20B	107.9
C7—C8—C9	122.90 (8)	C20—C21—C22	114.80 (8)
C7—C8—H8A	118.6	C20—C21—H21A	108.6
C9—C8—H8A	118.6	C22—C21—H21A	108.6
O2—C9—C8	122.21 (8)	C20—C21—H21B	108.6
O2—C9—C10	119.06 (7)	C22—C21—H21B	108.6
C8—C9—C10	118.72 (7)	H21A—C21—H21B	107.5
C15—C10—C11	117.94 (8)	C21—C22—C23	112.95 (7)
C15—C10—C9	124.00 (7)	C21—C22—H22A	109.0
C11—C10—C9	118.06 (7)	C23—C22—H22A	109.0
C12—C11—C10	121.04 (8)	C21—C22—H22B	109.0
C12—C11—H11A	119.5	C23—C22—H22B	109.0
C10—C11—H11A	119.5	H22A—C22—H22B	107.8
C11—C12—C13	120.15 (8)	C24—C23—C22	113.85 (8)
C11—C12—H12A	119.9	C24—C23—H23A	108.8
C13—C12—H12A	119.9	C22—C23—H23A	108.8
O3—C13—C14	125.19 (8)	C24—C23—H23B	108.8
O3—C13—C12	114.85 (8)	C22—C23—H23B	108.8
C14—C13—C12	119.96 (8)	H23A—C23—H23B	107.7
C15—C14—C13	119.15 (8)	C23—C24—C25	113.04 (8)
C15—C14—H14A	120.4	C23—C24—H24A	109.0
C13—C14—H14A	120.4	C25—C24—H24A	109.0
C14—C15—C10	121.76 (8)	C23—C24—H24B	109.0
C14—C15—H15A	119.1	C25—C24—H24B	109.0
C10—C15—H15A	119.1	H24A—C24—H24B	107.8
O3—C16—C17	104.98 (7)	C24—C25—H25A	109.5
O3—C16—H16A	110.8	C24—C25—H25B	109.5
C17—C16—H16A	110.8	H25A—C25—H25B	109.5
O3—C16—H16B	110.8	C24—C25—H25C	109.5
C17—C16—H16B	110.8	H25A—C25—H25C	109.5
H16A—C16—H16B	108.8	H25B—C25—H25C	109.5
C16—C17—C18	114.77 (8)		
			
C6—C1—C2—C3	0.50 (15)	C9—C10—C11—C12	179.29 (7)
C1—C2—C3—C4	−0.91 (16)	C10—C11—C12—C13	−0.32 (13)
C2—C3—C4—C5	0.14 (15)	C16—O3—C13—C14	4.73 (13)
C3—C4—C5—O1	−178.52 (9)	C16—O3—C13—C12	−175.05 (8)
C3—C4—C5—C6	1.05 (14)	C11—C12—C13—O3	−179.37 (8)
C2—C1—C6—C5	0.65 (14)	C11—C12—C13—C14	0.84 (13)
C2—C1—C6—C7	179.81 (9)	O3—C13—C14—C15	179.56 (8)
O1—C5—C6—C1	178.17 (8)	C12—C13—C14—C15	−0.67 (13)
C4—C5—C6—C1	−1.42 (13)	C13—C14—C15—C10	−0.01 (13)
O1—C5—C6—C7	−1.02 (12)	C11—C10—C15—C14	0.51 (13)
C4—C5—C6—C7	179.39 (8)	C9—C10—C15—C14	−179.10 (8)
C1—C6—C7—C8	4.99 (14)	C13—O3—C16—C17	−179.02 (7)
C5—C6—C7—C8	−175.86 (9)	O3—C16—C17—C18	−175.00 (7)
C6—C7—C8—C9	179.01 (8)	C16—C17—C18—C19	174.72 (8)
C7—C8—C9—O2	−6.40 (14)	C17—C18—C19—C20	174.57 (8)
C7—C8—C9—C10	173.90 (8)	C18—C19—C20—C21	−176.48 (7)
O2—C9—C10—C15	173.99 (8)	C19—C20—C21—C22	176.53 (8)
C8—C9—C10—C15	−6.30 (12)	C20—C21—C22—C23	−172.41 (8)
O2—C9—C10—C11	−5.62 (12)	C21—C22—C23—C24	−177.98 (8)
C8—C9—C10—C11	174.09 (7)	C22—C23—C24—C25	−172.02 (9)
C15—C10—C11—C12	−0.34 (13)		

### Hydrogen-bond geometry (Å, º)

Cg1 is the centroid of the C10–C15 ring.

**Table:**

*D*—H···*A*	*D*—H	H···*A*	*D*···*A*	*D*—H···*A*
O1—H1*O*1···O2^i^	0.939 (18)	1.789 (18)	2.6867 (12)	159.0 (16)
C7—H7*A*···O1^i^	0.93	2.31	3.2169 (13)	164
C22—H22*A*···*Cg*1^ii^	0.97	2.85	3.6887 (12)	146

Symmetry codes: (i) −*x*+2, −*y*+1, −*z*+1; (ii) *x*, *y*, *z*−1.
